# Risk factors for tuberculosis treatment failure among pulmonary tuberculosis patients in four health regions of Burkina Faso, 2009: case control study

**DOI:** 10.11604/pamj.2015.21.152.4827

**Published:** 2015-06-24

**Authors:** Bernard Sawadogo, Khin San Tint, Mufuta Tshimanga, Lazarus Kuonza, Laurent Ouedraogo

**Affiliations:** 1 South Africa Field Epidemiology and Laboratory Training Programme, South Africa; 2 School of Health System and Public health: University of Pretoria, South Africa; 3 University of Ouagadougou, Burkina Faso

**Keywords:** Pulmonary tuberculosis, treatment failure, risk factors

## Abstract

**Introduction:**

In Burkina Faso, the tuberculosis (TB) treatment failure rate increased from 2.5% in 2000 to 8.3% in 2006. The risk factors for TB treatment failure in the country are not well known. The study aims to determine the risk factors for treatment failure among pulmonary tuberculosis patients in four health region of Burkina Faso and to recommend appropriate interventions.

**Methods:**

A case control study was conducted among pulmonary TB patients who began TB treatment in 2009. A case was any patient who remained smear-positive at fifth month of TB treatment and a control was a patient who tested smear-negative at fifth month of treatment. A structured questionnaire was administered to one hundred cases and one hundred controls to collect information on exposure factors. Odds ratio were calculated using bivariate and multivariate analysis to determine the association between exposures and outcome.

**Results:**

Multivariate analysis showed that independent risk factors for TB treatment failure were fail to take TB drugs for more than 14 consecutive days (OR = 18.53; 95% CI:4.56 - 75.22), sputum smear-positive at two months of treatment (OR = 11.52; 95%CI:5.18-25.60), existence of comorbidity (OR = 5.74; 95%CI:1.69-19.44), and use of traditional medicines or herbs (OR = 2.97; 95%CI:1.12-7.85).

**Conclusion:**

Early identification of patients with the above risk factors for intense case management will improve TB treatment outcome. Patient with smear positive at 2nd^nd^ month of treatment require more intense follow-up, and involving traditional healers who provide traditional medicines or herbs in the educational programme on TB are required. The national referral laboratory capacity needs to be strengthened to do drug susceptibility testing and routine drug monitoring on cases of non conversion at 2^nd^ month of treatment.

## Introduction

In Burkina Faso, the incidence of TB, all forms, increased from 198/100 000 in 2000 to 241/100 000 population in 2004 before declining at 226/100 000 population in 2007 [1]. Of a total of 14 227 (96/100 000 population) new smear positive TB estimated by WHO [2] in 2007, only 2 614 (18%) were detected and notified. Of these new smear positive cases, 73% have been successfully treated. These are below the target of global TB control that is at least 70% case detection rate and 85% of treatment success rate [3]. Progress has been achieved in the follow-up of patients in recent years, with the default rate declining from 16.3% in 2000 to 5.6% in 2007 [4] . However the treatment success rate for new smear positive reached only 73% in 2006 because of high death rate (12%) and treatment failure rate (8%). The treatment failure rate increased from 4.1% in 2003 to 8.3% in 2006 before declining to 7% in 2007 [3]. Four of the 13 Health Regions of Burkina Faso notified 50% of treatment failure rate [3]. Although the number of patients who failed to TB-treatment is not high, the treatment failure rate is an important problem because of the possibility that these patients could harbor resistant Mycobacterium tuberculosis bacilli. The treatment failure is a health and economic burden as the patient remains a source of infection in the community and it may lead to the development of multidrug resistance, apart from the indirect economic burden attributed to absence from work and inability to work. There is very limited information on factors leading to TB treatment failure in Burkina Faso. Knowledge of factors associated with TB treatment failure could assist healthcare workers in identifying the particular personal stressors that inhibit compliance with TB treatment, as well as provider-level impediments to achieving treatment completion. This study aims to identify these factors associated with TB treatment failure in four health regions where treatment failure is reported. It is anticipated that the findings of this investigation will be used to develop an intervention site that will pilot the use of innovative case management approaches to reduce TB treatment failure rate, ensure treatment completion among pulmonary TB patients and prevent TB multidrug resistance.

Possible reasons for treatment failure in patients receiving appropriate regimens include [4, 5]: non adherence to the drug regimen, drug resistance, malabsorption of drugs, laboratory error, and extreme biological variation in response. In Finland a cohort study of risk factors for poor TB treatment outcome found that the significant risk factors included: male sex, older age, non-HIV related immunosuppression, previous history of TB, and pause in treatment [6]. In Brazil, researchers found that treatment delay, previous TB treatment, lifestyle factors, illiteracy and excessive alcohol consumption, and not having received family support were associated with treatment failure [7]. In Cameroon, a study report that age above or equal to 40 years and bacillary of 3+ or pre-treatment sputum smear were significantly associated with non conversion of sputum smear at the end of two months of treatment. Also persistent positive smear at the end of two months of treatment were significantly associated with unfavorable treatment outcomes [8]. An earlier case-control study in Egypt aiming at investigating predictors of treatment failure found that non-compliance to treatment, deficient health education to the patient, poor patient knowledge regarding the disease and diabetes mellitus as co-morbid condition were significant risk factors [9]. In Nigeria, a cohort of sputum smear-positive pulmonary tuberculosis patients at the initiation of therapy who were followed up to the end of treatment at eighth month reported that male gender and poor knowledge of tuberculosis predicted poor treatment outcome [10]. In Burkina Faso, a study conducted in the referral TB Centre (National Tuberculosis Centre in Ouagadougou) with 110 patients that had experienced failure, relapse, and treatment abandonment for at least 1 month observed a prevalence of 67.4% *M. Tuberculosis* drug resistance [11]. When studying risk factors for Multidrug-Resistant (MDR) TB in four centres in Burkina Faso, TB known contact, previous treatment, were risk factors significantly associated with MDR-TB [12]. The study objectives were: to determine the factors associated with TB treatment failure among pulmonary TB patients in four health regions in Burkina Faso, 2009; to recommend intervention project based on identified factors associated in order to reduce TB treatment failure.

## Methods

Pulmonary TB is defined as having two or more positive sputum smear examinations for Acid-Fast Bacilli; cured: patient who had completed full anti-TB treatment and was sputum smear negative after 5 months of treatment; treatment failure: sputum smear remained or became positive after 5 months of anti-TB treatment after treatment initiation; treatment delay: time interval between TB diagnosis and initiation of anti-TB drugs is more than 14 days; failure to take TB drug: who failed to take TB drug for 14 days or more; failure to collect TB drugs from health center: were considered failure to collect TB drug from health center when the medicine had been stock of out; having underlying condition: patients who declared having chronic condition such as liver disease, kidney disease, HIV/aids; having family member who take care: patients who have a family member who help him to feed, transport and remind him to take his TB drug. The North, North-Centre, South-Centre and Central-Plateau health regions have 431 primary health care centres where suspected TB are investigated and 27 TB diagnosis and treatment centres where sputum smear examinations are done and treatment given to TB patients [4]. The study was carried out in all 27 TB Centres of these four health regions.

A case-control study was conducted. The study population included pulmonary TB patients in the North, North-Centre, South-Centre and Central-Plateau health regions of Burkina Faso in 2009. The cases were pulmonary TB patients (hospitalized or not) who began TB treatment in 2009 and remained smear-positive, or became smear-positive again, after 5 months of treatment and the controls were pulmonary TB patients (hospitalized or not) who began TB treatment in 2009 and became smear-negative after 5 months of treatment. For each case of pulmonary TB, we chose one control from the same TB diagnosis and treatment centre and the same period of treatment initiation (within 30 days). Cases and controls were not matched for any suspected confounding factor. The cases were selected from the TB diagnosis and treatment register starting from 1^st^ January 2009. The controls were also selected from the same register as the cases. For each case, a control was the first patient cured following the case in the register. The interviewers recorded the physical address of the patients and sought them for interviews. In the case of refusal, the participant (case or control) was replaced by the following patient for each group. For the sample size, we used the rule of thump and 10 observations are required per factor under study [13, 14]. Our study looked at 10 variables, given a sample size of at least 100 patients for each group. Any patient who met the following three criteria was eligible for selection: testing smear-positive at the initiation of the treatment between 1^st^ January 2009 and December 2009 in the four health regions of Burkina Faso; being on TB treatment between 1^st^ January 2009 and December 2009 in the four health regions of Burkina Faso; having sputum smear examination result after 5 or 6 months of the treatment between 1^st^ January 2009 and December 2009 in the four health region of Burkina Faso. Any TB patient who died, defaulted or transferred within the study period was excluded as well as not meeting the inclusion criteria.

Data were collected by face-to-face interview and by review of the laboratory record register using a structured interviewer-administered questionnaire. The questionnaire was developed and approved by the study supervisor and the University of Pretoria Ethics committee. It was then piloted at Ouahigouya TB diagnosis and treatment centre in Burkina Faso during the month of August 2010, after the approval from the Secretary General of Ministry of health of Burkina Faso. The purpose of the pilot was to determine the feasibility of the sampling, whether the question can measure the variables of the study objectives, and the flow and the appropriate wordings of questions contained in the data collection tool. The questionnaire was standardised before data collection (see Annexure 1). Patients' interviews were carried out by health workers working at the TB diagnosis and treatment centres. The interviews were conducted at the patient's residence in a relaxed and conducive atmosphere. Health workers involved in the study were trained in interview techniques and supervised by the investigator. The variables that were considered in this study included characteristics of the patient (Age, Sex), patient related factors (having a member of family taking care of patient, receive support from the family such as food, money and transport, failure to visit TB centre to get TB drug, failure to take TB drug, sputum smear examination result at the initiation of treatment, after 2 months and 5 months of treatment, existence of underlying condition) and drug related factors (history of previous TB treatment, use of traditional medicines or herbs, history of TB drug side effects, treatment delay). During the supervision, we reviewed every completed questionnaire, ensuring that all the questions had been asked and all the questionnaire instructions had been followed. If necessary, questionnaires that were incomplete or incorrectly completed were given back to the interviewer and the appropriate section re-administered. A telephone call was also made daily to know the number of questionnaires filled. All questionnaires were checked as soon as possible after they are returned. Where information was missing or unclear, the interviewer was asked to resolve the query. This field supervision and check was particularly useful in filling gaps in the information and reconciling inconsistencies. In addition, it enabled the investigator to identify interviewers who were continuing to make the same mistakes and to provide those interviewers with necessary help or feedback to improve their work. Random selections of 8-10% of interviews were quality checked through second round interview by the investigator. Checks involved confirming the responses of the respective patient that surveyors had actually conducted as well as confirming key data items collected in the survey. The research proposal was submitted to the University of Pretoria Ethics Committee and to Burkina Faso Ethics Committee for Research in Health. Ethical approval was granted during the month of August 2010. Permission to use laboratory TB data and administer the questionnaire in the four health regions of Burkina Faso was also requested in writing from the Ministry of health of Burkina Faso. Permission was granted to conduct the study by the Secretary General of Ministry of health of Burkina Faso (see annexure 4). TB is strongly associated with HIV/AIDS which stigmatizes and discriminates TB patients. TB has become difficult to discuss in public. The identity of the respondents was not recorded. Confidentiality was important and patient information was not made available to persons outside the study team. Respondents were further assured that no personal-identifiers would be used for publication. The Informed consent form was signed by responders and assent if patient was less than 18 years. The questionnaires were kept in a locked cupboard and the data kept in a laptop protected by a password. The participants received information about TB and those who were found not adhering to TB treatment got health education and advices. The outcome variable was TB treatment failures. This variable is categorical (0, 1) and exposure variables were continuous (age) and categorical (age group, sex of patient, sputum smear examination result at the initiation of treatment, after 2 and 5 months of treatment, underlying condition, failed to visit TB center to get TB drug, failed to take TB drug, family member take care (family member stays with the patient at hospital), received support from family, history of previous TB treatment, use of traditional medicines or herbs, history of TB drug side effects and treatment delay).

For stratified analysis we performed the following steps: we performed crude analysis by calculating crude OR for each factor with confidence interval and p-value; we stratified the data by potential confounders and effect modifiers (age group and sex). we calculated stratum-specific OR; age group < 45 years and 45 years and older were considered; cut off 45 years was chosen to be able to compare with other studies; we evaluated for effect modification by comparing stratum-specific OR; If effect modification is present (stratum-specific OR differ from one another), we stopped and reported stratum-specific OR; If effect modification is absent (stratum-specific OR similar), we assessed and control for confounding as necessary by comparing the stratum-specific OR with the crude OR. If the crude OR value does not fall within the range between the smallest and the largest stratum-specific OR, confounding is present; We then calculated the weight average of stratum-specific OR (Mantel-Haenszel summary OR) and compared the adjusted OR with the crude OR to see if they are "appreciably different". Multivariate analysis to measure the effect of multiple risk factors on TB treatment failure was also performed. All risk factors with p-value < 0.2 in bivariate analysis were introduced in the model. Binary outcome variable or dependent variable was case status (0/1; 0 for control and 1 for case). The binary independent variable or exposure variables were: sex of patient (0/1), sputum-smear positive after 2 months (yes/no), existence of underlying condition (yes/no), failure to visit TB centre to get TB drug (yes/no), failure to take TB drug (yes/no), family member take care (yes/no), received support from family (yes/no), history of previous TB treatment (yes/no), use of traditional medicines or herbs (yes/no) and history of TB drug side effect (yes/no). The logistic regression model was: Logit (Casestatus) = sex of patient + sputum-smear positive after 2 months + existence of underlying condition + failure to visit TB centre to get TB drug + failure to take TB drug + family member take care + received support from family + history of previous TB treatment + use of traditional medicines or herbs + history of TB drug side effect. We began the process by starting with simple model and building up until we reached what we consider our best and final model, taking into consideration significant variable with p-value less than 0.05.

## Results

The majority of patients (60%) were from North health region. Median age was 39.5 years (ranged 18-77 years) for cases and 38.5 years (ranged 18-82 years) for controls. As show in Table 1, cases and controls were comparable for age except age group of less than 25 years. Cases had more male when controls had almost the same between male and female. Cases and controls were also comparable for marital status, education level and occupation.

**Table 1:** Characteristics of TB patients (cases and controls) in four health regions of Burkina Faso, Jan-Dec 2009

Characteristics	Cases n (%)	Controls n (%)
**Age group**		
< 25 years	2 (2%)	12 (12%)
25-44 years	59 (59%)	54 (54%)
45-54 years	21 (21%)	17 (17%)
> 55 years	18 (18%)	17 (17%)
**Sex of patients**		
Female	27 (27%)	44 (44%)
Male	73 (73%)	56 (56%)

### Bivariate analysis

In Bivariate analysis, in Table 2, sex was significantly associated with TB treatment failure. Cases were two times more likely to be male (p = 0.006) than controls. Being less than 45 years old is protective. However it is not statistically significant.

**Table 2:** TB treatment failure by age group and sex among cases and controls in four health regions of Burkina Faso, Jan-Dec 2009

Factors	Cases n (col%)	Control n (col%)	OR	95% CI	p-value
**Age**					
< 45 years	61 (61%)	66 (66%)	0.80	0.45 - 1.43	0.23
45+ years	39 (39%)	34 (34%)			
**Sex of patient**					
Male	73 (73%)	56 (56%)	2.12	1.17 - 3.84	0.006
Female	27 (27%)	44 (44%)			

OR= Odd ratio CI = Confidence Interval

### Patient related factors

Table 3 shows patient related factors. Among all these factors, having sputum-smear positive after 2 months of treatment (OR= 8.90; 95%CI: 4.65-17.01), having an underlying condition (OR= 4.67; 95%CI: 1.82-12.07), failure to collect TB drugs from TB centre (OR= 4.40; 95%:1.2-16.14), failure to take TB drugs (OR = 17.41; 95%CI:5.13-58.98), were significantly associated with TB treatment failure (p < 0.05). Underlying conditions reported by patients included liver disease, kidney disease and other diseases such as HIV, high blood pressure, and asthma.

**Table 3:** Patient related factors associated with TB treatment failure in four health regions of Burkina Faso, Jan-Dec 2009

Factors	Cases n (col%)	Control n (col%)	OR	95%CI	p-value
**Sputum smear positive at 2**^**nd**^ **month**					
Yes	80 (80%)	31 (31%)	8.90	4.65-17.01	<0.001
No	20 (20%)	69 (69%)			
**Underlying condition**					
Yes	23 (23%)	6 (6%)	4.67	1.82-12.07	<0.001
No	77 (77%)	94 (94%)			
**Failure to collect TB drug from TB center**					
Yes	12(12%)	3(3%)	4.40	1.2-16.14	0.014
No	88 (88%)	97(97%)			
**Failure to take TB drug**					
Yes	35 (35%)	3 (3%)	17.41	5.13- 58.98	< 0.001
No	65 (65%)	97 (97%)			
**Family member take care of patient**					
Yes	82(82%)	89(89%)	0.56	0.25-1.26	0.08
No	18(18%)	11(11%)			
**Receives support from family**					
Yes	80(80%)	86(86%)	0.65	0.30-1.37	0.13
No	20(20%)	14(14%)			

OR= Odd ratio CI = Confidence Interval

### Drug related factors

Table 4 shows that among TB drugs related factors evaluated, having history of previous TB treatment (OR = 6.15; 95%CI: 1.73-21.87), use of traditional medicines or herbs (OR = 4.55; 95%CI:2.15-9.61), history of TB drug side effects (OR = 2.52; 95%CI:1.37-4.65) were significantly associated with TB treatment failure (p < 0.05) . The average time between diagnosis and treatment starting is 3.5 days ranges from 0 to 30 days for cases and 2.5 days ranges from 0 to 12 days for controls. The delayed in starting treatment is 14 days. TB drugs' side effects reported by TB patients (cases and controls) were nausea/vomiting, dizziness, abdominal cramps, rash, and diarrhoea. For those who used traditional medicines or herbs 8% of cases versus 0% of controls stopped using TB drug, 5% of cases versus 3% of controls had vomiting and 9% of cases versus 1% of controls had diarrhoea.

**Table 4:** Drug related factors associated with TB treatment failure in four health regions of Burkina Faso, Jan-Dec 2009

Factors	Cases n (col%)	Controls n (col%)	OR	95%CI	Mid p exact
History of previous TB treatment					
Yes	16 (16%)	3 (3%)	6.15	1.73 - 21.87	<0.001
No	84 (84%)	97 (97%)			
Use of traditional medicines/herbs					
Yes	36 (36%)	11 (11%)	4.55	2.15 - 9.61	< 0.001
No	64 (64%)	89 (89%)			
History of TB drug Side effect					
Yes	77 (77%)	57 (57%)	2.52	1.37 - 4.65	0.001
No	23 (23%)	43 (43%)			
Treatment delay					
< 14 days	98 (98%)	99 (99%)	0.49	0.04 - 5.54	0.5
14+ days	02 (2%)	01 (01%)			

### Stratify analysis

Factors associated with TB treatment failure were stratified by age group and sex to assess for possible confounding and effect modification (seeTable 5, Table 6).

**Table 5:** Factors associated with TB treatment failure stratified by age-group in four health regions of Burkina Faso, Jan-Dec 2009

Factors	Age group	cases	controls	Crude OR (95%CI)	Stratum specific OR (95%CI)	MH Adjusted OR (95%CI)
History of Previous TB treatment	**< 45 years**			6.15 (1.73-21.87)	12.74 (1.57-102.84)	NA
	Yes	10	1			
	No	51	65			
	**45+ years**				2.90 (0.54-15.49)	
	Yes	6	2			
	No	33	32			
Failure to collect TB drug from TB centre	**< 45 years**			4.40 (1.20-16.14)	8.42 (1.00-70.63)	NA
	Yes	7	1			
	No	54	65			
	**45+ years**				2.35 (0.42-13.00)	
	Yes	5	2			
	No	34	32			
Sputum-smear positive after 2 months of treatment	**< 45 years**			8.90 (4.65 - 17.01)	12.61 (5.32 - 29.87)	NA
	Yes	51	19			
	No	10	47			
	**45+ years**				5.31 (1.94 - 14.53)	
	Yes	29	12			
	No	10	22			
Underlying condition	**< 45 years**			4.67 (1.81-12.07)	3.79 (1.15-12.50)	NA
	Yes	12	4			
	No	49	62			
	**45+ years**				6.28 (1.28-30.81)	
	Yes	11	2			
	No	28	32			
Failure to take TB drug	**< 45 years**			17.41 (5.13 - 58.98)	15.60 (3.46 - 70.34)	NA
	Yes	20	2			
	No	41	64			
	**45+ years**				20.62 (2.54- 166.99)	
	Yes	15	1			
	No	24	33			
Use of traditional medicine or herbs	**<45 years**			4.55 (2.15-9.61)	4.87 (1.80-13.19)	4.49 (2.12-9.52)
	Yes	20	6			
	No	41	60			
	**45+ years**				4.03 (1.28-12.66)	
	Yes	16	5			
	No	23	29			
History of TB drug side effects	**<45 years**			2.52 (1.37-4.65)	2.34 (1.10-4.95)	2.49 (1.35-4.60
	Yes	45	36			
	No	16	30			
	**45+ years**				2.82 (0.96-8.25)	
	Yes	32	21			
	No	7	13			

**Table 6:** Factors associated with TB treatment failure stratified by sex in four health regions of Burkina Faso, Jan-Dec 2009

Factors	Sex	Cases	Controls	Crude OR	Stratum specific OR	MH adjusted OR
Sputum-smear positive after 2^nd^month	**Male**			8.90 (4.65-17.01)	5.54 (2.54-12.08)	NA
	Yes	58	23			
	No	15	33			
	**Female**				19.80 (5.74-68.20)	
	Yes	22	8			
	No	5	36			
Failure to take TB drugs	**Male**			17.41 (5.13-58.98)	38.37 (5.02-292.74)	NA
	Yes	30	1			
	No	43	55			
	**Female**				4.77 (0.85-26.62)	
	Yes	5	2			
	No	22	42			
History of TB drugs side effects	**Male**			2.52 (1.37-4.65)	2.46 (1.17-5.16)	2.75 (1.46-5.18)
	Yes	54	30			
	No	19	26			
	**Female**				3.62 (1.06-12.29)	
	Yes	23	27			
	No	4	17			
Use of traditional medicines or herbs	**Male**			4.55 (2.15-9.61)	4.89 (1.85-12.91)	4.52 (2.11-9.68)
	Yes	27	6			
	No	46	50			
	**Female**				3.90 (1.14-13.31)	
	Yes	9	5			
	No	18	39			
Underlying condition	**Male**			4.67 (1.81-12.07)	5.36 (1.48-19.35)	4.77 (1.80-12.58)
	Yes	17	3			
	No	56	53			
	**Female**				3.90 (0.88-17.19)	
	Yes	6	3			
	No	21	41			
History of Previous TB treatment	**Male**			6.15 (1.73-21.87)	3.13 (0.83-11.83)	5.28 (1.54-18.03)
	Yes	11	3			
	No	62	53			
	**Female**				NA	
	Yes	5	0			
	No	22	44			
Failure to visit TB centre to get TB drugs	**Male**			4.40 (1.20-16.14)	NA	5.56 (1.35-22.77)
	Yes	9	0			
	No	64	56			
	**Female**				1.70 (0.31-9.14)	
	Yes	3	3			
	No	24	41			

### Age as effect modifier

Table 5 shows that aged less than 45 years had two times more likely to have sputum smear-positive after 2 months of treatment than aged 45 years and older. Age appear to be effect modifier to odd of sputum smear-positive at 2nd month of treatment. Among cases, those aged less than 45 years old were 4 times more likely to have been previously treated for TB than those aged 45 years and older. Age appear to be effect modifier. Cases aged 45 years and older have 65% times more likely to have other underlying condition than cases less than 45 years. Age appear to be effect modifier. Cases less than 45 years old have three times more likely to fail to visit TB centre to get TB drug than cases more than 45 years. Age is not effect modifier to use of traditional medicine or herbs, history of TB drug side effects and received support from family.

### Sex as effect modifier

Table 6 shows that among TB treatment failure, males were three times more likely to have sputum smear-positive after 2 months of treatment than females. The sex appears to be effect modifier. Also, males were eight times more likely to fail to take TB drugs than females. Age appears to interact with sputum smear result after 2 months of treatment, existence of underlying condition, history of previous TB treatment, failure to visit TB centre to get TB drug centre and the sex interacts with laboratory result positive after 2 month of treatment and fail to take TB drug.

### Multivariable analysis

Multivariable logistic regression analysis was performed to study the factors associated with TB treatment failure while controlling other possible factor associated (Table 7). Those independent factors, found to be significant by chi-squared test in bivariate analysis with p < 0.2, were introduced in the model: sex of patient, sputum-smear positive after 2 months, existence of underlying condition, failure to collect TB drug, failure to take TB drug, family member take care, received support from family, history of previous TB treatment, use of traditional medicines or herbs, history of TB drug side effect. The factors found to be significantly associated with TB treatment failure in the multivariable analysis were: failure to take TB drug (OR = 18.53; 95%CI: 4.56 - 75.22); sputum smear-positive after 2 months of treatment (OR = 11.52; 95%CI: 5.18-25.60); existence of underlying condition (OR = 5.74; 95%CI: 1.69 - 19.44); use of traditional medicines or herbs (OR = 2.97; 95%CI: 1.12 - 7.85).

**Table 7:** Multivariable analysis of factors associated with TB treatment failure (cases and controls) in four health regions of Burkina Faso, Jan-Dec 2009

Risk factors	OR	95%CI	P-value
Failure to take TB drug	18.53	4.56 - 75.22	<0.001
Sputum smear positive after 2 months of treatment	11.52	5.18 - 25.60	<0.001
Existence of Underlying condition	5.74	1.69 - 19.44	0.004
Use of traditional medicine/herbs	2.97	1.12 - 7.85	0.02
Sex of patient	1.19	0.54 - 2.65	0.65
Failure to collect TB drug	2.67	0.27 - 25.97	0.39
Family member take care	1.44	0.20 - 10.02	0.71
Received support from family	1.32	0.21 - 8.32	0.76
History of previous TB treatment	3.90	0.72 - 21.05	0.11
History of TB drug side effects	1.33	0.57 - 3.10	0.49

## Discussion

Cases were 11 times more likely to have sputum smear positive after 2 months of treatment than controls and this association increased in male. Studies have also reported that patients who fail to TB treatment are more likely to have sputum smear positive after 2 months of treatment [15–17]. Patients with positive cultures after 2 months of treatment should undergo careful evaluation to determine the cause [4]. Researchers from American Thoracic Society, CDC, and Infectious Diseases Society of America also reported that for patient who have positive cultures after 2 months of treatment and have not been receiving DOT, the most common reason of non conversion is non-adherence to the regimen [4]. Studies from China and from Thailand also reported relationship between a positive sputum examination at two months and treatment failure [18, 19]. Munoz in Ethiopia and Singla in India reported similar findings [20, 21]. These patients will require more intense follow-up throughout their treatment including the continuation phase. Cases were 5 times more likely to have other underlying condition (liver disease, kidney disease, high blood pressure, HIV) than controls. This odd to have underlying condition increased with age of patient. Also, the treatment of TB in patients with unstable or advanced liver disease is problematic for several reasons. Firstly, the likelihood of drug-induced hepatitis may be greater. Secondly, the implications of drug-induced hepatitis for patients with marginal hepatic reserve are potentially serious, even life-threatening [4]. Management of HIV-related TB is complex and requires expertise in the management of both HIV disease and tuberculosis as HIV-infected patients are often taking numerous medications, some of which interact with anti-tuberculosis [4]. Having underlying disease is associated with TB treatment failure and medical consultation should be provided for TB patients with underlying disease.

Independently the use of traditional medicines or herbs may lead to kidney and liver disease that were also associated with TB treatment failure, Traditional medicines or herbs may interact with the absorption and metabolism and, this inhibit the effect of TB drug. The association of traditional medicines or herbal use and TB treatment failure in our study is explained by the fact that usage of traditional medicines/herbs is three times more in cases than in control. Our findings are similar to Brouwer's who found that 73% of his patient stated that they did not improve or deteriorate on traditional treatment and herbal use [22]. Because of the large number of traditional healers who provide traditional medicines or herbs to patients in the community, some of whom are very influential; we believe it is important to involve traditional healers as stakeholders in educational activities of the national TB Control programme. Traditional healers need to be taught to recognize illness such as TB and its management with proven anti-tuberculosis treatment. More importantly, a population-based study could provide more definitive evidence.

In our study, cases were 18 times more likely to fail to take their TB medication. This odd is 8 times higher in males. Among cases, the most common reason for failing to take TB drugs, was that side effects were either too common or too much to deal with. Other reasons for failure to take TB drug were travelling trips, closure of health facilities during strikes and absence of health care workers to provide TB drugs. Our findings are similar to Morsy's in Egypt who reported that 8 missed doses are associated with an increase in the risk of treatment failure [9]. This case control study highlights the factors associated with TB treatment failure. These factors are: patients who failed to take TB drugs, patients who had underlying condition such as liver and kidney disease, patients who used traditional medicines or herbs, and patient who still have sputum smear positive after 2 months of treatment. Previous treatment guidelines have recommended that if the smear is positive after 2 months of treatment, continue the initial phase for an extra month before proceeding with the standard 4-month continuation phase. It is designed for low-resources settings that lack the ability to do culture or drug-susceptibility testing. These recommendations have been questioned in our study and in other studies [16–21], as the extra month of treatment before proceeding with the standard 4-month continuation phase does not guaranty treatment success at the end of the 6 month treatment. More studies need to be done to assess this recommendation. Moreover, the national referral laboratory capacity to do drug susceptibility testing and routine therapeutic drug monitoring earlier in case of non conversion after 2 months of treatment is very crucial.

Our study has some limitations: the time-lag between the TB treatment failure and the interview was around one year and might result in a recall bias. Also the interviews were conducted by health workers in TB diagnosis and treatment centres who knows previously the patients and this will lead that participants respond to particular questions with the answers that they consider to be most socially desirable (or least stigmatizing), rather than answering with complete honesty. The factors related to health care services were not investigated and may play a role in TB treatment failure.

## Conclusion

The independent risk factors for TB treatment failure identified are failing to take TB drugs for more than 14 days, having underlying diseases such as liver and kidney disease, usage of traditional medicines or herbs, and having sputum smear positive after 2 months of treatment. Factors related to health care services were not investigated and may also play a role in TB treatment failure. There is thus need to study health centre-specific challenges to high TB treatment success rate in our environment. Based on these findings we recommended the following: to improve the compliance to TB treatment, National TB Control Programme should train health workers in the TB centre regarding TB case management. This training should include supporting patients' care, treatment package, closer individual case management and treatment support particularly to patients who complain to drug side effect. To avoid incorrectly use of traditional medicines or herbs by TB patients, TB control coordinator in each district should involve traditional healer who provide these medicines in the educational activities regarding TB control. Traditional healer need to be taught to recognize TB and refer early patients to TB centre instead of given them traditional medicine or herbs. More study need to be done to provide evidence of use of traditional or herb regarding TB treatment. Health workers in TB Centre should be provided with clear guidelines on management of TB patient with underlying disease such as liver and kidney disease. These guidelines should include diagnostic of these underlying diseases, their treatment, the interaction with TB drugs, need of adjustment of TB drug doses, where and when to refer for medical consultation, etc. Training may improve the use of these guidelines. Patient with smear positive after 2 months of treatment require more intense follow-up throughout their treatment including the continuation phase. National referral laboratory capacity need to be strengthened to do drug susceptibility testing and routine therapeutic drug monitoring earlier in case of non conversion after 2 months of treatment.
